# Innate Immune Induction and Influenza Protection Elicited by a Response-Selective Agonist of Human C5a

**DOI:** 10.1371/journal.pone.0040303

**Published:** 2012-07-06

**Authors:** Sam D. Sanderson, Marilyn L. Thoman, Kornelia Kis, Elizabeth L. Virts, Edgar B. Herrera, Stephanie Widmann, Homero Sepulveda, Joy A. Phillips

**Affiliations:** 1 School of Allied Health Professions, University of Nebraska Medical Center, Omaha, Nebraska, United States of America; 2 Sidney Kimmel Cancer Center, San Diego, California, United States of America; 3 Biosciences Center, San Diego State University, San Diego, California, United States of America; 4 BD Biosciences, San Diego, California, United States of America; Mount Sinai School of Medicine, United States of America

## Abstract

The anaphylatoxin C5a is an especially potent mediator of both local and systemic inflammation. However, C5a also plays an essential role in mucosal host defense against bacterial, viral, and fungal infection. We have developed a response-selective agonist of human C5a, termed EP67, which retains the immunoenhancing activity of C5a at the expense of its inflammatory, anaphylagenic properties. EP67 insufflation results in the rapid induction of pulmonary cytokines and chemokines. This is followed by an influx of innate immune effector cells, including neutrophils, NK cells, and dendritic cells. EP67 exhibits both prophylactic and therapeutic protection when tested in a murine model of influenza A infection. Mice treated with EP67 within a twenty-four hour window of non-lethal infection were significantly protected from influenza-induced weight loss. Furthermore, EP67 delivered twenty-four hours after lethal infection completely blocked influenza-induced mortality (0% vs. 100% survival). Since protection based on innate immune induction is not restricted to any specific pathogen, EP67 may well prove equally efficacious against a wide variety of possible viral, bacterial, and fungal pathogens. Such a strategy could be used to stop the worldwide spread of emergent respiratory diseases, including but not limited to novel strains of influenza.

## Introduction

The primary role of the innate immune system is to provide an immediate line of defense against foreign pathogens [1], [2], [3]. Activation of innate immunity to pathogens typically involves release of acute-phase proteins including IL-6, IL-8 and TNF. This results in a rapid influx of PMNs (polymorphonuclear leukocytes, primarily neutrophils), natural killer (NK) cells, and macrophages to the site of infection. This innate response acts to contain the pathogen during development of the acquired immune response. Due to its rapid onset and non-specific nature, there is a great deal of interest in developing therapeutic agents that combat infection via innate immune induction. This could result in therapeutics able to protect against multiple, diverse pathogens, moving away from the current “one bug, one drug” system.

The complement system has been largely overlooked in the search for immunotherapeutic agents. Made up of a number of soluble inactive precursor proteins present throughout the body, the complement cascade can be activated by binding of complement components directly to the surface of a pathogen (the alternative complement pathway). Activation of the various complement components generates a series of small, pharmacologically active byproducts including the anaphylatoxin C5a, a 74-residue glycopolypeptide that activates cells via its ligation with surface-expressed C5a receptors (C5aR/CD88). Binding of C5a to its cognate receptor on myeloid lineage cells induces cytokines and chemokines involved in modulating host innate immunity, rapidly followed by chemotaxis of innate immune effector cells [4], [5]. CD88 is also expressed on inflammatory cells such as neutrophils. Binding of C5a to CD88 on these cell populations leads to both local and systemic inflammatory responses [6], [7], sequelia that would appear to obviate therapeutic exploration of the C5a pathway.

Despite the deleterious inflammatory processes associated with C5a, the interaction between C5a and the C5aR plays a critical, non-redundant role in mucosal host defense against bacterial, viral, and fungal infection [8], [9], [10], [11], [12]. This is particularly well-described in the case of influenza. Mice lacking C5 are extremely susceptible to influenza-induced mortality [13]. Following influenza infection, complement receptors and C5a are both upregulated in the upper respiratory tract [14], [15]. Blocking the C5aR during influenza infection severely decreases both the frequency and absolute numbers of influenza-specific CD8^+^ T cells, along with lowering overall CTL activity and the IFN-gamma response to immunodominant viral epitopes [10], [16]. Separation of this C5a-induced protection from any deleterious C5a induced inflammatory processes could yield a powerful therapeutic agent.

The biologically active region of human C5a is confined to the C-terminal ten residues (i.e., C5a_65–74_, ISHKDMQLGR). Our laboratory has developed a conformationally biased agonist of C5a_65–74_ that retains the immunostimulatory capacity of human C5a but does not possess its anaphylactic capacity. This response-selective agonist is known as EP67 and has the sequence Tyr-Ser-Phe-Lys-Asp-Met-Pro-(N-methylLeu)-D-Ala-Arg or YSFKDMP(MeL)aR. EP67 is one of a series of synthetic C5a agonist peptides bearing unique conformational features that are well accommodated by the C5aR expressed on APCs, but not on inflammatory PMN [17], [18], [19], [20]. Previous studies have exploited this separation of C5a-like immune induction from inflammation to develop vaccine adjuvants with efficacy in multiple species [18], [21], [22], [23], [24], [25], [26], [27]. In the course of these studies, it was determined that EP67 induces release of IL-6 and TNF from CD88-positive APC populations [26], [27]. IL-6 and TNF are critical inducers of the acute phase response to infection, leading to the hypothesis that EP67 could act as a solo therapeutic agent.

The purpose of this study was to test the impact of EP67 on induction of innate immunity. Our results show that EP67 induces a rapid burst of cytokines and chemokines in the airways followed by the appearance of multiple innate immune effector cell populations. This induction of innate immunity is protective against influenza-induced morbidity and mortality, even when EP67 treatment occurs one day after infection with a lethal dose of influenza. These results indicate that EP67, previously used as a vaccine adjuvant, can function as a stand-alone therapeutic agent with efficacy against established respiratory infection.

## Materials and Methods

### Ethics Statement

All animal experimental protocols were approved by the Institutional Animal Care and Use Committees at the Sidney Kimmel Cancer Center and at San Diego State University prior to initiation of experiments. Animals were given free access to food and water and were cared for according to guidelines set by the American Association for Laboratory Animal Care.

### Animals

C57BL/6 mice were bred in house. Male and female mice between 12 and 20 weeks old were used. For EP67 insufflation, mice were anesthetized with isofluorane and 30 µl of EP67 was applied to the nares. Each animal was watched to ensure that the entire volume was aspirated. Control animals were given 30 µl of intranasal saline. Animals were monitored until they could right themselves (<1 minute) and returned to their cage. At no time was any distress observed in response to EP67 insufflation. Preliminary experiments confirmed that the pulmonary response to EP67 was not due to any contaminants from peptide synthesis by comparing the response to EP67 with that of a control peptide composed of the same amino acids scrambled into a different order (scEP67; ([MeL]RMYKPaFDS). Insufflation of mice with the scrambled EP67 peptide did not induce any discernable pulmonary response (Figure S1) compared to insufflation with an equal volume of saline or no treatment at all. This is in agreement with our previously published data showing that the specific mechanism underlying EP67 activity is CD88 ligation, to the exclusion of any non-specific stimulation [26], [28].

### Influenza Infection

For influenza infection studies, mice were anesthetized with either isofluorane by inhalation or ketamine/xylazine by injection and then infected with influenza virus strain A/Puerto Rico/8/34 H1N1 (A/PR/8) purchased from Charles River Labs Avian Vaccine Services (North Franklin, CT). The HA titer of the purchased virus was 1∶655,360/ml, and the EID_50_ titer of 10^9.3^/ml. Preliminary testing in our laboratory indicated that the MLD_50_ was 10^3.4^ EID (data not shown). In the experiments shown, we employed a non-lethal dose of 0.33×MLD_50_, (10^2.9^ EID) and a lethal dose of 3.3×MLD_50_ (10^3.9^ EID) per mouse, delivered in a volume of 30 µl as above. For non-lethal infection, mice were treated with 45 µg EP67, and for lethal infection mice were treated with 240 µg EP67. Body weight and general appearance of infected mice was monitored daily following infection. Any animal that lost 30% starting body weight was sacrificed and the infection counted as lethal (reviewed in [29]).

### Peptide Synthesis

EP67 (YSFKDMP(MeL)aR) and scrambled EP67 ([MeL]RMYKPaFDS), were synthesized by standard Fmoc (9-fluorenyl-methoxycarbonyl) solid-phase methods on a pre-loaded Arg Wang resin by sequential coupling of the HBTU (2-(1Hbenzotriazole-1-yl-1,1,3,3-tetramethyluronium hexafluorophosphate) esters of each amino acid as described [30]. EP67 was cleaved from the resin via standard acidolysis with a TFA (trifluoroacetic acid) cocktail containing phenol (5%), water (2%), and triisopropylsilane (2%) as scavengers. EP67 was purified by analytical and preparative reverse-phase HPLC in C18-bonded silica columns with 0.1% TFA as the running buffer (solvent A) and 60% acetonitrile in 0.1% TFA (solvent B) as the eluant, and characterized by molecular mass (M+H)^+^ with electrospray mass spectrometry. EP67: M_calc_ = 1240, (M +1H)^+^ = 1241.3910, (M +2H)^2+^ = 621.1760; scrambled EP67: M_calc_ = 1240, (M +1H)^+^ = 1241.5267, (M +2H)^2+^ = 621.2435.

### Bronchoalveolar Lavage

To collect BAL fluid and cells, animals were exposed to a high concentration of isofluorane until respiration ceased, and then exsanguinated by severing the abdominal aorta. The trachea was exposed, cannulated with an 18-gauge, ½ inch needle connected to a 1-ml syringe, and the lungs were flushed once with 1 ml of PBS. The BAL samples were spun for ten minutes at 1200 RPM, and the fluid was removed from the cell pellet and frozen for later analysis. The remaining BAL cells were resuspended for staining and flow cytometric analysis. BAL cell counts were performed using an Accuri C6 flow cytometer according to the manufacturer’s instructions.

### Flow Cytometric Analysis

Antibodies specific for Ly5.2-FITC, MHCII (I-A/I-E-PE), Gr-1(Ly-6G)-PE and B220-PE, were from BD Pharmingen, San Diego, CA. Antibodies specific for CD80-PE, CD86-PE, NK1.1-PE, CD14-PE, CD11b-AlexaFluor647, and anti-CD11c-PECy7 were from eBioscience (San Diego, CA). Following removal of the BAL fluid for cytokine analysis, BAL cells were suspended in RPMI without phenol red containing 5% FBS. Cells were stained with various combinations of fluorescent antibodies for 30 minutes on ice, then washed and analyzed using an Accuri C6 flow cytometer. FACS data was analyzed using GateLogic software (Inivai, Menton Victoria, Australia). All samples were stained with anti-Ly5.2 to eliminate contaminating red blood cells as well as any non-hematopoetic lung cells. BAL cells from individual mice were not pooled prior to staining and analysis. All FACS studies were repeated 2–5 times. Staining specificity was confirmed using a fluorochrome-matched irrelevant staining antibody.

### Pulmonary Viral Burden

Three days after infection, pulmonary viral titer was monitored by quantitative real-time PCR (qPCR) of the viral matrix RNA. For qPCR, total RNA was prepared by homogenizing infected lung tissue in TRIZOL (Life Technologies) with 1 mm beads using a Bullet Blender (NextAdvance) at setting 8 for five minutes. One hundred nanograms total RNA from each lung was analyzed in duplicate 20 µl reactions using the RNA-To-CT 1-Step Taqman kit (Life Technologies) and the influenza matrix primer and probe set described by Spackman [31]. The viral load was calculated using a standard curve generated from RNA isolated from uninfected lung to which known numbers of influenza virions had been added prior to RNA isolation. The 50% tissue culture infectious dose (TCID_50_), was determined exactly as described [32] using the data analysis method of Reed and Muench [32].

### Cytokine and Chemokine Analysis

Analysis of BAL cytokines was performed using the Cytokine Bead Array from BD Pharmingen (San Diego, CA) as described [33]. These assays had a sensitivity limit of 10 pg/ml [34]. Individual BAL samples from three mice were analyzed for each time point. After the initial studies, the concentration of IL-1ß, IL-12p70, and IL-10 in the BAL fluid samples was further analyzed using CBA Enhanced Sensitivity Flex Sets. This extends the sensitivity of the assay down to 274 fg/ml. Samples were run on a FACSArray, and analysis was performed using FCAP Array software. The exception to this was analysis of IFN-ß, which was performed by sandwich ELISA as described [35]. Briefly, plates were coated with monoclonal hamster anti-mouse IFN-ß (BioLegend, San Diego, CA) and a standard curve was constructed using recombinant murine IFN-ß (Aviva Systems Biology, San Diego, CA). Polyclonal rabbit anti-mouse IFN-ß (VWR) used as a secondary antibody followed by biotinylated donkey anti-rabbit (Jackson ImmunoResearch) as the detecting antibody. The ELISA was developed using avidin-alkaline phosphatase and p-nitrophenylphosphate (Sigma) as a substrate and read at 405 nm (background subtract at 490 nm on a BioRad Microplate reader.

### Humoral Response to Influenza

Inactivated influenza A/PR/8 bound directly to the ELISA plates served as the antigen. The antibody subclass analysis examined the influenza specific IgG1, IgG2b, and IgG2c response. The gene encoding IgG2a is deleted in C57BL/6 mice. This strain expresses IgG2c, which shares 84% homology with IgG2a [36]. Total anti-mouse IgG, IgG1, and IgG2b were from L Laboratories, and anti-mouse IgG2c was from Beckman Coulter.

## Results

### EP67 Induces Cytokine and Chemokine Production in the Airway

**Figure 1:**
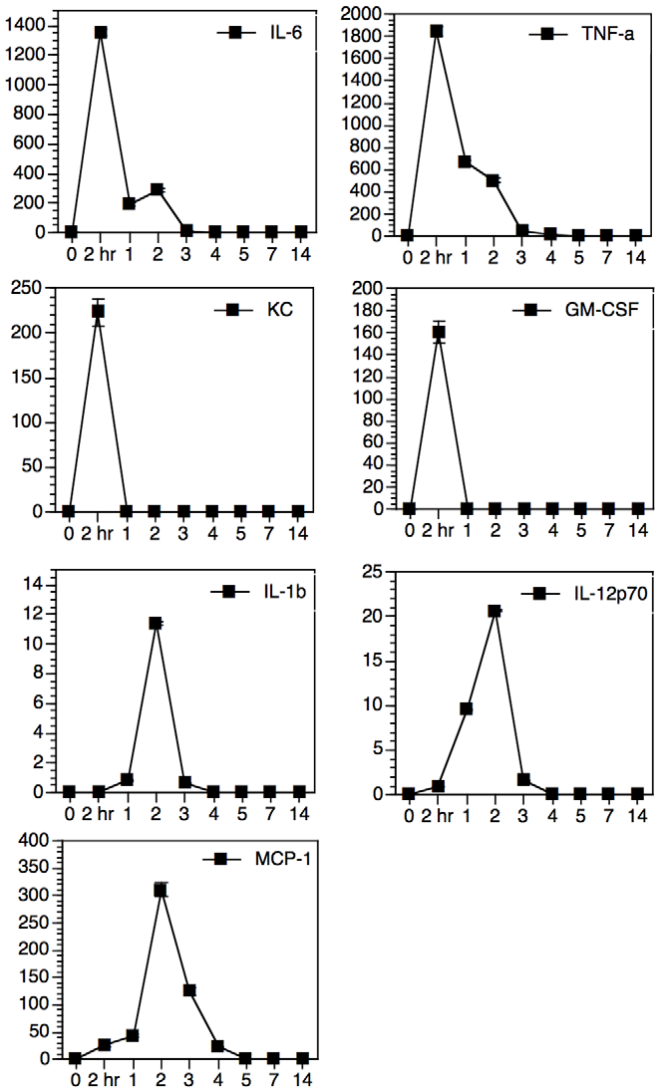
EP67 induced cytokines and chemokines. Mice were insufflated with 30 micrograms EP67 in a total volume of 30 microliters, and then sacrificed at various time points between two hours and fourteen days later. Cytokine analysis was performed as described in Materials and Methods. Three individual samples (not pooled) were tested for each time point. For each cytokine graph, the X-axis is time in days (except for the initial two hour time point) after EP67 insufflation. The Y-axis is specific cytokine concentration in pg/ml. Data is displayed as group average ± SEM for each time point.

Mice were insufflated with EP67 and sacrificed between 2 hours and 3 weeks later. Lungs were lavaged one time with 1 ml PBS, and the BAL fluid was analyzed for a panel of cytokines using a bead-based assay. EP67 induced two waves of cytokine/chemokine induction, with distinct expression peaks present at two hours and two days after EP67 treatment (Figure 1). Two hours after EP67 insufflation, IL-6, TNF, GM-CSF, and KC/CXCL1 were all detected at increased concentrations in the BAL fluid. By one day post EP67, the concentration of all these mediators had decreased precipitously, with both KC/CXCL1and GM-CSF dropping below the limit of detection (10 pg/ml). The concentration of TNF decreased daily until indistinguishable from background on day five. In contrast, the local concentration of IL-6 dropped sharply at twenty-four hours, but then increased again at forty-eight hours before returning to background levels by day four post-treatment. This second peak of IL-6 matched the second wave of cytokine expression. This second peak was composed of IL-6, IL-1ß, IL-12p70, and MCP-1/CCL2. At the 2-hour time point, IL-12p70 and MCP-1/CCL2 were present at low concentrations and IL-1ß was indistinguishable from background; however, all three were detectable by twenty-four hours and displayed a peak concentration at forty-eight hours post-treatment. IL-1ß and IL-12p70 returned to background levels by day 4 and MCP-1/CCL2 by day five. At no time point were concentrations of either IL-10 or IFN-ß in the BAL fluid samples above background. Real-time PCR analysis of IFN-ß mRNA was performed at several time points following EP67 insufflation; however, we were unable to confirm any increase in expression of the IFN-ß message.

### EP67 Alters the Cellular Makeup of the Airway

**Figure 2:**
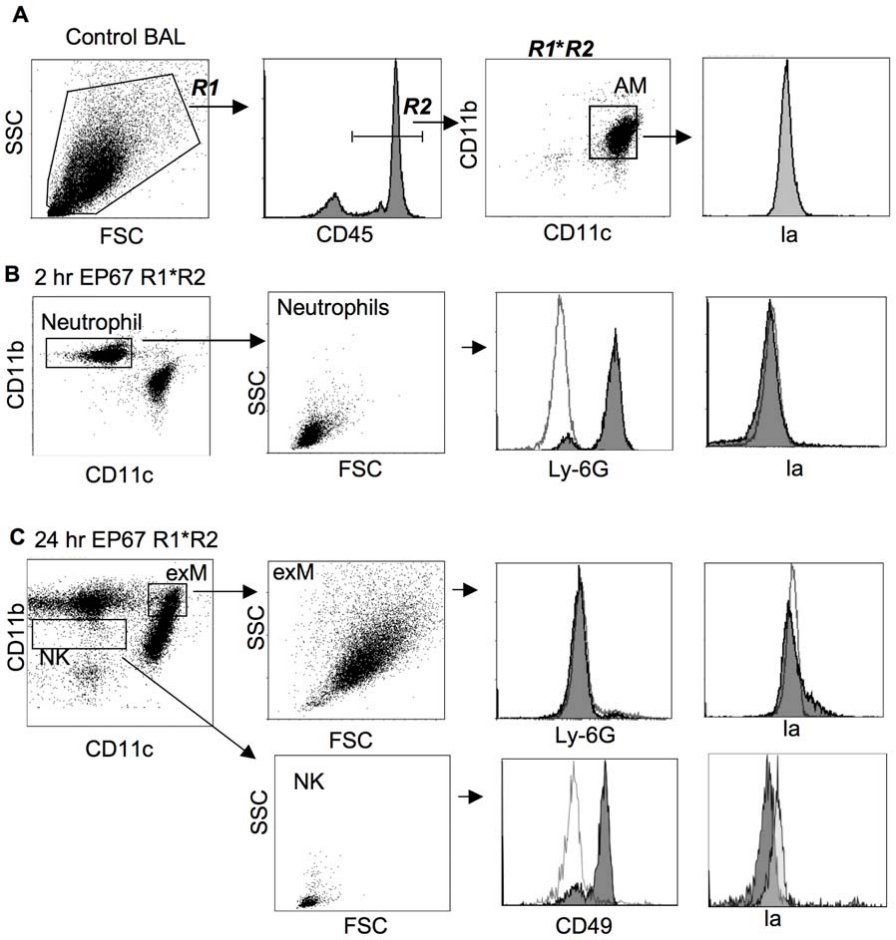
Innate effector cells in the BAL following EP67 insufflation. Mice were insufflated with EP67 as in Figure 1. BAL cells were isolated either two hours or one day later, then stained with fluorescent antibodies and analysed by FACS. FACS plots shown are from individual mice representative of at least 10 mice per time point. For surface marker analysis, empty histograms represent negative control stains and filled histogram overlay represents the stain listed on the X-axis. FSC: forward scatter (size); SSC: side scatter. A. Gating strategy for BAL cell analysis, using a non-EP67 stimulated control mouse. All samples were initially gated by size and side scatter and stained with CD45.2 to exclude any non-hematopoetic cells or red blood cells from analysis. All CD45 positive cells were stained with CD11c and CD11b to identify discrete cell populations. The identity of individual cell populations was confirmed using additional surface stains, as shown. B. BAL cells isolated two hours after EP67 insufflation. Boxed cells indicate cell populations not present as a large percentage of BAL prior to EP67 insufflation. C. BAL cell analysis one day after EP67 insufflation. Representative staining data is shown (10–30 mice per time point).

The cytokines present two hours after EP67 are classic mediators of the acute phase response, during which effector cells are recruited to the site of tissue damage or infection. Therefore, we examined the cell populations in the airways following EP67 insufflation. Animals were sacrificed at various time points ranging from two hours through three weeks following EP67 treatment as above and cells present in the BAL were analyzed by FACS. The gating and analysis strategy is shown in Figure 2A. A preliminary gate was drawn using the FSC/SSC plot to include all cells present in the BAL. Positive staining with CD45, which is expressed on all cells of hematopoietic origin except red blood cells (RBC), was used as a specific gate to exclude RBCs or non-leukocyte cell populations.

Consistent with previously published reports [37], [38], the major cell population identified in the airways of control mice was alveolar macrophages (AM). These cells are defined as FSC^hi^CD11b^−^CD11c^+^ with negligible levels of surface MHC II. Two hours after EP67 insufflation, a population of CD11b^+^CD11c^−^ were identified in the BAL (Figure 2B). Forward scatter analysis showed that these were small cells and additional surface staining showed they expressed the neutrophil-specific marker Ly6G. In control mice, CD11b^+^CD11c^−^ neutrophils represented only 0.5–5% of total BAL cells. Two hours after EP67 insufflation, however, neutrophils made up about 60% of the cells present in the BAL.

Twenty-four hours after EP67 treatment, two additional cell populations were detected in the BAL (Fig. 2C). The first of these were CD11b^+^CD11c^+^, large cells that were negative for Ly-6G and low/neg for MHC II, matching the phenotype of exudate macrophages (exMac). In control mice, cells of this phenotype represented only 1–3% of total BAL cells. Twenty-four hours after EP67 treatment, however, this percentage increased to 15% of the total BAL cell population. The second cell type identified at 24 hours was negative for CD11b, CD11c, and Ly-6G. These were very small cells that stained positive for the natural killer cell marker CD49. In unstimulated BAL samples, NK cells are difficult to detect with any degree of certainty. Following EP67 stimulation, the NK cells represented 1% of the total BAL population.

**Figure 3:**
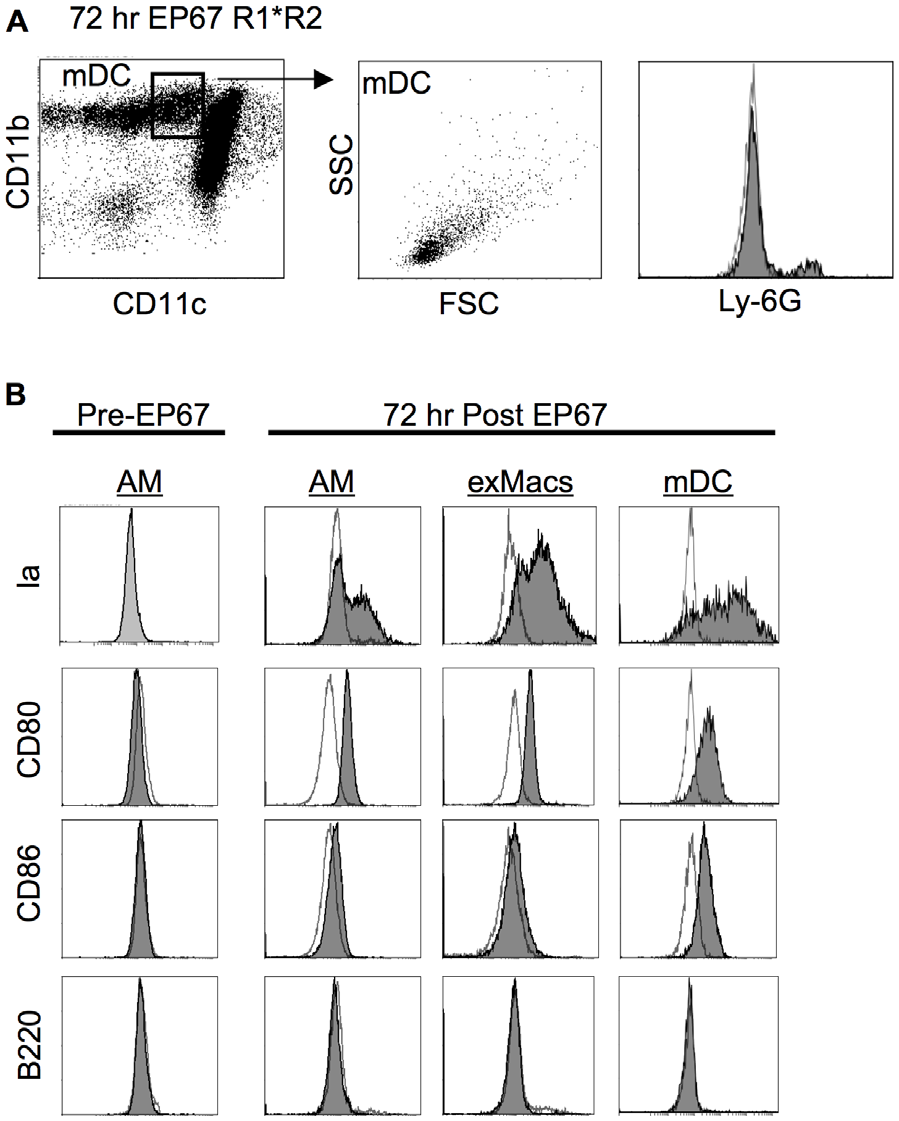
Antigen presenting cell populations in the BAL following EP67 insufflation. Mice were insufflated with EP67 as in Figure 2, and BAL analysis was carried out three days later. A. Analysis of myeloid dendritic cells. Isolation, staining, and analysis was carried out three days after EP67 insufflation as in Figure 2. B. Surface marker analysis on antigen presenting cell populations following EP67 insufflation. Negative control stains are shown as empty histograms; the filled histogram overlay is the specific stain listed on the left. Representative staining data is shown (10–20 mice per time point).

Three days after EP67 treatment, a third population of cells was identified in the BAL (Figure 3A). These were CD11b^hi^CD11c^mid^ cells. They displayed less surface CD11c than either the exMac or the AM populations and slightly more surface CD11b than the neutrophils. Although difficult to cleanly discriminate from the neutrophils based on CD11b and CD11c expression, this population of cells was larger, displayed less side- scatter, and were negative for the neutrophil marker Ly6G. They were also high positive for expression of MHC II, which confirmed their identity as myeloid dendritic cells (mDC). The mDCs were clearly identifiable as a separate population 72 hours after EP67 treatment.

Cell surface expression of MHC II and co-stimulatory markers CD80 and CD86 on the BAL cell populations induced by EP67 insufflation are shown in Figure 3B. Again consistent with previous reports, AM from untreated mice did not display surface MHC II or the costimulatory markers CD80 or CD86 [39]. However, a large percentage of AM expressed cell surface MHC II three days after EP67 treatment. The AM had also upregulated surface expression of CD80 but not CD86. Expression of B220 was also negative. This same panel of surface markers was displayed by the exMac population. The mDCs identified 3 days after EP67 were highly positive for MHC II, CD80 and CD86. The absence of B220 on the mDC confirmed their myeloid origin, distinguishing them from the B220-positive plasmacytoid DCs. Neutrophils and NK cells exhibited no induction of MHC II, CD80, CD86, or B220 (not shown).

**Figure 4:**
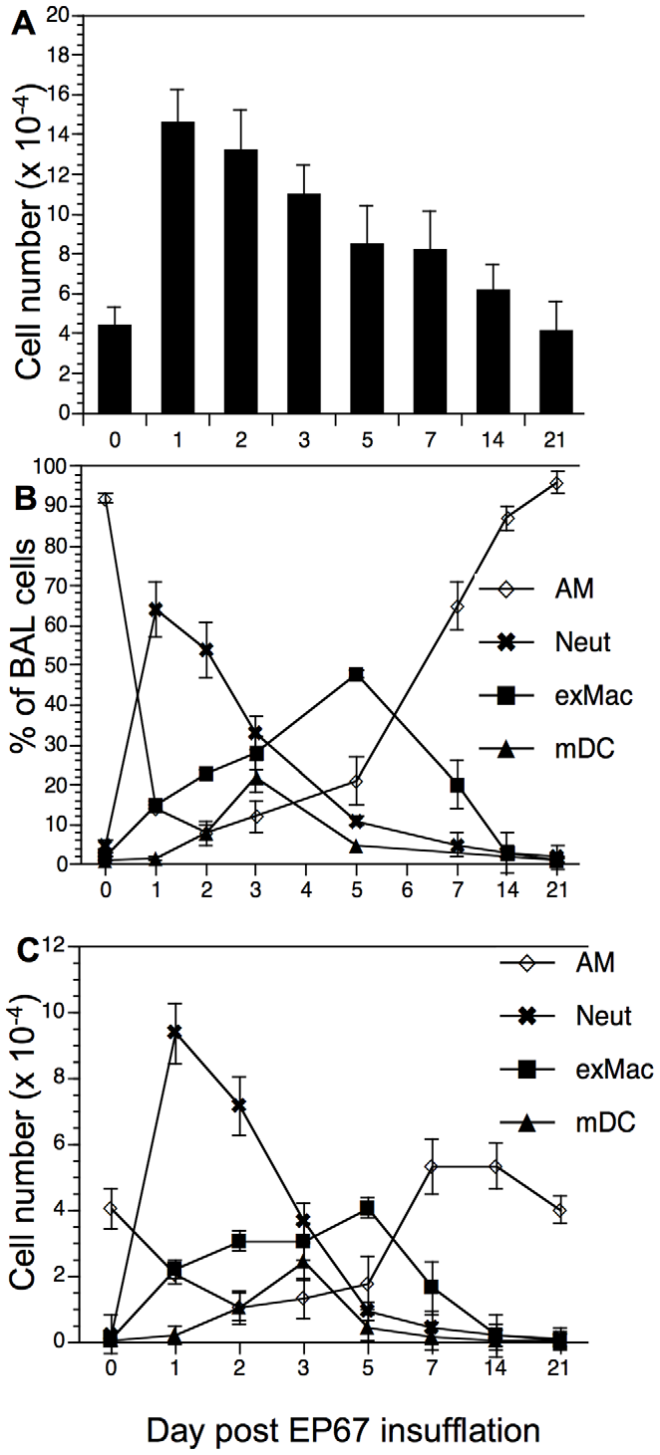
Kinetics of innate effector cells in the airways following EP67 insufflation. X-axis is time in days following EP67 insufflation. A. Absolute cell number following EP67 insufflation. B. Subpopulations of BAL cells as % of BAL cell population. C. Total cell number of individual BAL cell subpopulations. AM: open triangles; Neutrophils: **x**; exMacs: closed squares; mDC: closed triangles. NK cells are not shown, as they never represented more than 1% of the total BAL population. Results are shown as mean ± SD.

This dynamic appearance of innate immune effector cells into the airway is reflected in the numbers of cells present in the BAL over time (Figure 4). Control mice had an average of 4×10^4^ cells recovered from the airway. The total number of BAL cells peaked at 1.5×10^5^ one day after EP67 treatment and declined daily thereafter. Neutrophils achieve their peak one day after EP67 treatment both in terms of total number of cells and as a percentage of the BAL cell population. Neutrophils declined rapidly after day three and returned to background within one week. The exMacs in the BAL increased gradually over five days, at which time they represented the majority of BAL cells and then dropped back to control levels by day fourteen. The mDC population showed little change on day 1, but increased in percentage and total number on day two before reaching a peak on day 3, at which time they represented 20% of BAL cells. Both the number and percentage of the mDC dropped precipitously back to control levels by day five.

### EP67-mediated Cell Flux is Dose-dependent

**Figure 5:**
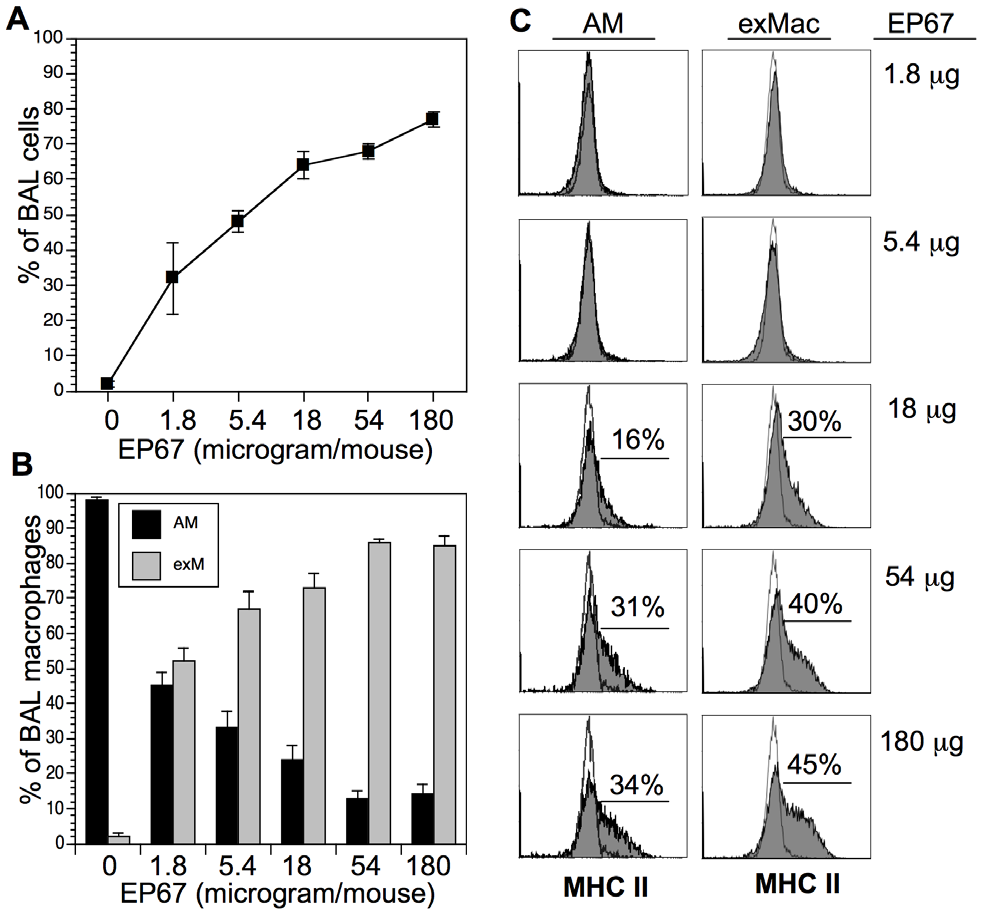
Dose-response profile following EP67 insufflation. Mice (3–5/group) were insufflated with varying doses of EP67, and BAL cells were analyzed one day later as in Figure 2. A. Neutrophils as a percentage of total BAL cells (y-axis) vs. increased dose of EP67 (x-axis). Results are shown as group mean ± SD. B. Ratio of alveolar macrophages to exudate macrophages based on expression of CD11b (y-axis) vs. increased dose of EP67 (x-axis). C. Upregulation of MHC II on AM and exMac with escalating doses of EP67. Empty histogram represents negative control stain; filled histogram represents MHC II expression. Numbers in histogram represent increased % of cells staining vs. negative control stain. Dose of EP67 is shown on the far right. There was no clear increase in MHCII expression at the lowest doses of EP67; therefore, there is no listed percentage in those histograms.

Preliminary observations following EP67 insufflation indicated that the appearance of innate immune effectors in the BAL was dose dependent. To investigate this further, mice were insufflated with various doses of EP67 ranging from 1.8 µg to 180 µg and then sacrificed one day later for analysis of the BAL cell populations using CD11c, CD11b, and MHC II as above. As shown in Figure 5A the EP67-mediated influx of neutrophils is dose-dependent. The neutrophil influx shows no evidence of either a bell-shaped dose response or of maximum induction over the range of doses tested. Within the macrophage population (Figure 5B), the ratio of exMac-to-AMs also increased with increasing doses of EP67. Prior to EP67 insufflation, exMac represent about 2% of the airway macrophage population. One day after insufflation with the lowest dose of EP67 tested, exMacs made up about 52% of the total macrophage population. This percentage increased with dosage up to 54 µg/mouse. Higher doses did not further increase the percentage of exMacs relative to the AM population.

We also examined the effect of increasing the EP67 dosage on the expression of MHCII by the macrophage populations (Figure 5C). The lowest doses examined did not induce a measurable increase in expression of MHC II compared to that on AMs from untreated mice. Upregulation of surface MHC II expression is not detected until animals are insufflated with a dose of 18 µg EP67. Both the AM and exMac populations displayed increased expression of cell-surface MHCII at the higher EP67 doses.

### EP67 Induces a Protective Anti-influenza Response

**Figure 6:**
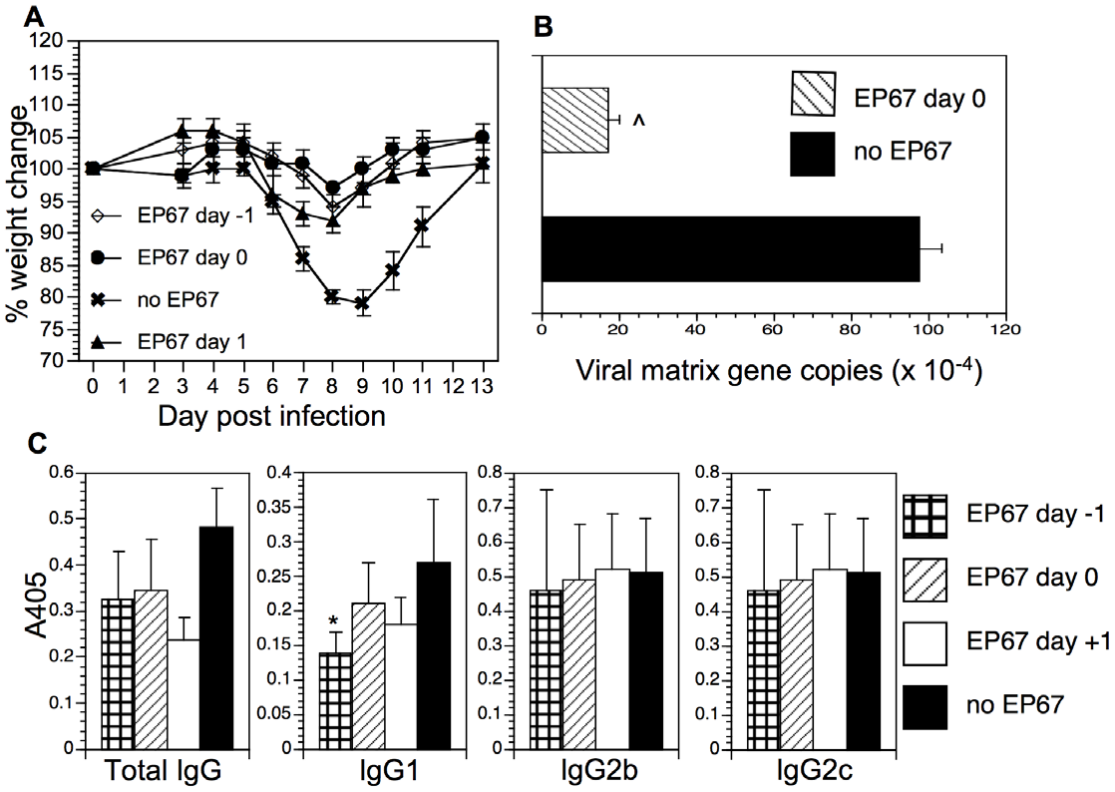
EP67 displays protection against influenza-induced morbidity. Four groups of mice were infected with a non-lethal dose of influenza (0.33×MLD_50_) as described in Materials and Methods. Three groups were treated one time with EP67, either the day before infection (EP67 day −1), at the time of infection (EP67 day 0), or the day after infection (EP67 day 1). Mice were weighed daily. A. Weight change following influenza infection. Group average weight change is shown as mean ± SEM. Day 8 weight loss results are statistically significant (using a two-tailed Student’s t-test) as reported in the text. One experiment is shown; experiment was repeated twice. B. Viral load in lungs was measured using quantitative RT-PCR of the influenza matrix gene. Lung RNA was isolated three days after infection. Results are shown as matrix gene copies per 100 ng RNA. (^∧^p<0. 0005, two-tailed Student’s t-test). C. ELISA analysis of anti-influenza antibodies. Individual serum samples were analyzed for total IgG, IgG1, IgG2b, and IgG2c distribution. Results are displayed as mean ± SEM. The total IgG response shown is from the 1∶10,000 dilution of serum. The subclass responses shown are from the 1∶1000 serum dilution. *p<0.05, two-tailed Student’s t-test.

The appearance of neutrophils, NK cells, and exMacs in the alveolar space is reminiscent of the innate immune response to influenza infection [40]. To determine if EP67 could provide protection from influenza infection, mice were infected with a non-lethal dose of influenza A/PR/8 on day 0. Individual cohorts were insufflated one time with EP67 on day −1, 0, 1, 2, or 3 relative to the day of infection. Animals were weighed daily and an untreated control cohort was included as a positive control for influenza-induced weight loss. The results are shown in Figure 6. The cohort that was infected with influenza but did not receive EP67 treatment (**x**, no EP67) showed the standard pattern of weight loss and regain over the 2 weeks after infection. This cohort lost an average of 20% starting body weight on day 8 post-infection, with all weight regained by day 13 post-infection. Mice treated with EP67 within a one-day window of infection (i.e., day −1, day 0, or day +1 relative to time of infection) were significantly protected from influenza-induced weight loss on day eight post infection. Mice treated with EP67 either one day prior to infection, the day of infection, or one day after infection lost an average of 6% (p<0.001), 3% (p<0.0001), and 8% (p<0.05), respectively of their starting body weight. Treatment with EP67 later than 1 day after infection was not associated with significant protection from influenza induced weight loss (not shown). Protection was associated with a significant reduction in viral RNA measured three days after infection (p<0. 0005).

Following return to starting weight of all cohorts, serum was tested for the presence of anti-influenza antibodies by ELISA. The significant reduction in pulmonary viral burden following EP67 treatment was not associated with a decrease in the humoral anti-influenza response (Figure 6C). Although the total anti-influenza IgG response was lower in all groups that exhibited significant protection from influenza-induced weight loss, this decrease did not reach statistical significance. Analysis of the IgG subtypes showed that the concentration of IgG1 was also decreased in the protected cohorts, but that this decrease was only statistically significant in one of the treatment conditions. The concentrations of IgG2b and IgG2c showed no decrease relative to the infected cohort in any of the treatment groups.

### EP67 Induces Innate Immunity in the Presence of Influenza Infection

**Figure 7:**
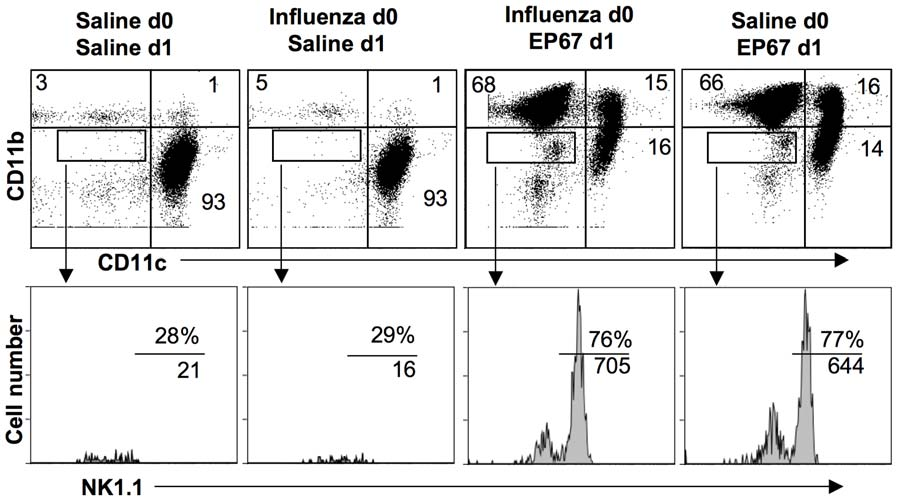
The innate immune activation capacity of EP67 is not abrogated by established influenza infection. Mice were insufflated with either saline or a non-lethal dose of influenza as in Figure 6. One day after infection, mice were insufflated with EP67 or saline. All mice were sacrificed 48 hours after influenza infection, 24 hours after EP67 treatment. BAL cells were analysed as in Figure 2. Top row: BAL cells were stained for CD11c and CD11b. Percentage of cells in quadrant is shown. Bottom row: Cells in box were analyzed for expression of NK1.1. Percentage of NK1.1 positive cells in the analysis box is shown above the bar, and absolute number of cells is shown below the bar. Representative results are shown; experiment was repeated twice.

Initiation of innate immunity is normally delayed for up to 48 hours after infection with influenza A due to the viral suppression of host innate immunity [41], [42], [43]. Therefore, we examined the EP67 response in the context of an established influenza A infection. Mice were infected with a non-lethal dose of A/PR/8 and then treated via insufflation 1 day later with EP67 or saline. Animals were sacrificed two days after infection (one day after EP67 or saline treatment). As shown in Figure 7, the phenotype of BAL cells from control mice which were neither infected nor treated with EP67 matched that of mice infected with influenza A/PR/8 and treated with saline 1 day later. Under both conditions (i.e., unmanipulated vs infected) the majority of the cells present in the BAL were AM with a low percentage of CD11b^+^ neutrophils (similar to the results in Figure 2A) and few scattered cells in the NK gate. In contrast, mice infected with influenza and treated one day later with EP67 exhibited a robust influx of neutrophils, NK cells, and exMacs. The phenotype and percentage of the innate effectors induced by EP67 in the presence of established influenza infection was similar to that seen in mice treated with EP67 in the absence of an established influenza infection.

### Delayed EP67 Treatment Protects from Lethal Influenza Infection

**Figure 8:**
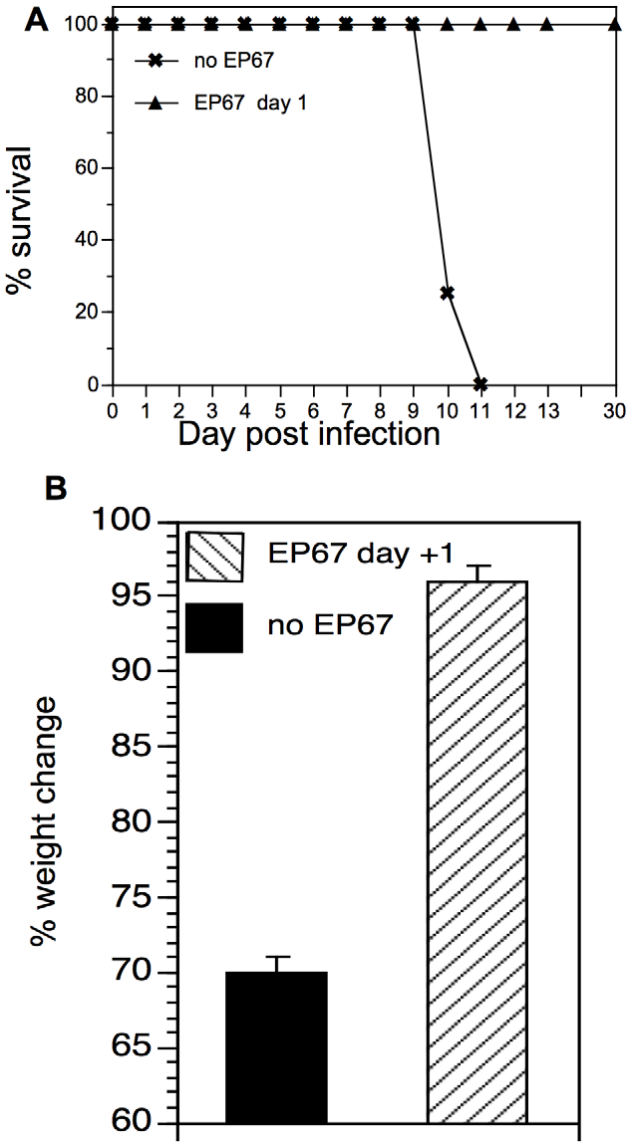
EP67 prevents morbidity and mortality following lethal influenza infection. Mice (five mice/group) were infected with a lethal dose of influenza (3.3×MLD_50_) as described in Materials and Methods. One day later, groups were insufflated with either EP67 (240 µg/mouse) or saline. Mice were sacrificed when weight loss reached 30% of starting body weight, and the infection counted as lethal. A. Survival curve. B. Group average weight loss.

Mice were infected with a lethal dose of influenza A and treated one day later with EP67 (240 µg/mouse) or saline. Weight and appearance were monitored daily. Mice that lost ≥30% of starting body weight and displayed typical symptoms of illness (piloerection, hunching, lethargy) were sacrificed and the infection scored as lethal. Using these criteria, all of the control mice succumbed to the infection by day eleven post-infection. In marked contrast, 100% of mice exposed to the same lethal dose but treated one day later with EP67 survived the infection (Figure 8). During the course of the experiment, the EP67-treated animals exhibited no signs of hunching, lethargy, or piloerection. Treatment with EP67 one day following lethal infection induced significant protection from influenza-induced weight loss (p<0.0001).

## Discussion

The ability to induce innate immunity safely has long been a goal for treating viral, bacterial, and fungal infections. Increasing resistance to pulmonary infection following non-specific immune stimulation has been recognized for over thirty years [44], [45]. Such a strategy requires development of therapeutic entities able to induce various elements of innate immunity with little/no adverse inflammatory side effects.

The anaphylatoxin C5a is pharmacologically active byproduct of the complement cascade. C5a acts to bring inflammatory cells to the site of tissue injury/infection and to subsequently activate their effector responses. These functions are dependant upon binding of C5a to the cell-surface receptor CD88 (reviewed in [46], [47]. Like native C5a, EP67 function is critically dependent upon CD88 ligation [26], [28]. However, although it is able to activate antigen presenting cells, EP67 neither binds to nor signals through the CD88 surface receptor on inflammatory PMNs [19]. Therefore, insuffulation of EP67 induces the acute phase proteins TNF and IL-6 in the absence of accompanying neutrophil stimulation [19], [26], [27], [48].

The current study shows that delivery of EP67 to the airways induces a potent anti-viral response characterized by rapid cytokine induction and sequential influx of innate effector cell populations. A single insufflated dose of EP67 was sufficient to initiate a cascade of molecular and cellular events covering a period of days. The resultant innate immune response provided 100% protection following infection with a lethal dose of influenza. This EP67-induced cascade of events displayed the essential characteristics and kinetics expected for a protective innate response to infection. The cytokines and chemokines detected in the airways within two hours of EP67 treatment (TNF, IL-6, GM-CSF, and KC/CXCL1) are all important mediators of direct antiviral protection. TNF is by itself a robust inhibitor of influenza A replication [49], [50], [51]. During an active infection, TNF also enhances the recruitment of leukocytes to the site of infection and activates innate immune responses [49], [50], [51]. IL-6 exhibits pleiotropic effects, including the activation of NK cells and macrophages and stimulation of T cell differentiation during influenza infection [50], [52]. GM-CSF enhances survival, proliferation, maturation, and differentiation of myeloid cells. GM-CSF also blocks mortality from lethal influenza infection and is critical to alveolar growth and repair [53], [54]. The chemokine KC/CXCL1 plays a critical role in preventing bacterial pneumonia after influenza infection [55].

In addition to direct anti-viral and protective functions, the cytokines and chemokines induced by EP67 are all associated with chemotaxis of innate immune effector cell populations. A single administration of EP67 induces the recruitment of neutrophils within two hours. Neutrophils are one of the first inflammatory cell type to appear in large numbers during acute infection [2], [56]. Neutrophils may contribute to innate immune protection during the early stages of influenza infection, although their requirement is debated [57], [58]. Although neutrophils exhibit chemotaxis directly to native C5a, EP67 is devoid of C5a-like engagement of CD88-bearing PMNs [19]. The cytokines and chemokines detected two hours after EP67 treatment are all associated with neutrophil chemotaxis [59], and both TNF and GM-CSF act to increase neutrophil survival times in tissue from hours to days [56]. The peak neutrophil response (both total number and percentage of BAL) occurred twenty-four hours after EP67 treatment. Stimulated neutrophils produce a large number of proinflammatory mediators, including TNF and IL-1β [56]. If EP67 were directly activating the neutrophil population, it would be anticipated that the local concentration of these cytokines would greatly increase at or near the peak of the neutrophil response. However, the local concentration of TNF and IL-1ß show no correlation to the kinetics of neutrophil influx and disappearance. This argues against neutrophils as the cellular source of the airway cytokines, underscoring an important therapeutic attribute of EP67; i.e., its ability to recruit neutrophils for the initial assault on an infection, but no capacity to activate them directly once recruited. In the absence of a wide-spread infection, this avoids induction of an unnecessary or overly-aggressive inflammatory outcome.

Within one day of EP67 treatment, both NK cells and exMacs are present in the airways. Three days after EP67, mDCs can be detected as a large percentage of the airway cells. The involvement of these cell types in the clearance of various airway infections is well established (reviewed in [2]). NK cells lyse virally-infected cells, acting to contain viral infection during maturation of the acquired immune response. NK cells also play a critical role in activation of the CTL response to influenza [60]. Macrophages phagocytize pathogens, and loss of the exMac population is associated with highly increased pulmonary influenza titers and a decrease in the subsequent T cell response [40]. The pulmonary DCs initiate the adaptive immune response by presenting antigen to naïve T lymphocytes [2]. EP67 also enhanced expression of MHC II and costimulatory surface markers on the APC populations (i.e., AM, exMac, and mDC) in the airways in a dose dependant fashion. An increased antigen-presenting capacity secondary to this observed maturation of APC populations is consistent with our previous work showing that that EP67 (as well as the earlier sister analogue EP54) are effective adjuvants in both young and aged mice [18], [21], [22], [23], [24], [25], [26], [27].

In the normal lung, the AM and the bronchial epithelium (BE) are continually exposed to the outside world during respiration. Both cell types can rapidly release cytokines in response to inflammation/infection. Furthermore, both AM and BE express the C5aR CD88 and are able to secrete cytokines in response to both natural C5a and EP67 or its earlier sister analogs ([61] and Sanderson, unpublished data). It is thus quite likely that the AM and the bronchial epithelium both contribute to the immediate cytokine response following EP67 insufflation. The cellular source of those cytokines that peak two days after EP67 treatment (IL-1ß, IL-12p70, and MCP-1, as well as the second peak of IL-6) is less clear. By this late time point, it is unlikely that any EP67 remains in the airways. This second wave of mediators is most likely derived from continual activation of the initiating responder cells and/or to maturation of different innate effector cell populations in the airways. For example, mDCs can release multiple chemokines over several days [62], with the later phases biased toward recruitment of monocytes and T-lymphocytes. This would be consistent with the presence of IL-12p70, considered a “Th1-type” cytokine, as well as MCP-1, a monocyte chemoattractant. The second peak of IL-6 deserves mention. IL-6 acts as both a pro-inflammatory and an anti-inflammatory cytokine. The latter function is critical to the resolution of the innate immune response (reviewed in [63]). The mechanisms involved include antagonism of the TNF response and induction of neutrophil apoptosis. The rapid loss of neutrophils three days after EP67 treatment corresponds both with the limits of apoptosis protection induced by TNF and GM-CSF as well as the second local increase in the IL-6 concentration.

The EP67-induced release of TNF and IL-6 followed by an influx of innate immune effector cells resembles the innate immune response to influenza infection (reviewed in [2], [64]. However, the innate immune response to influenza infection does not normally commence for a matter of days due to antagonism of the antiviral type 1 IFN pathway by the NS-1, PB1-F2, and PB2 viral proteins [41], [42], [43]. Suppression acts at the level of the infected cell, delaying initiation of the innate immune response during the early stages of viral replication and spread. Because of this both mice and humans experience unconstrained viral replication during the earliest time points following infection. In the absence of treatment, the endogenous anti-viral immune response is generally initiated two days after infection, with effector cells appearing in the lungs a day later [2], [65]. Not until initiation of the innate immune response do viral titers begin to diminish. Early treatment with EP67 induced a robust response even in the presence of an established infection, initiating an anti-viral response up to two days earlier than in the untreated animals. This led to a significant decrease in the pulmonary viral burden, reflected in protection from weight loss and death.

Although early treatment was clearly protective, it was unsurprising that a single dose of EP67 delivered two or three days after infection shows no clear therapeutic benefit. By this time post-infection, the endogenous innate response has been initiated. In the case of uncomplicated (non-lethal) infection, there may be no therapeutic benefit to increasing the already-initiated innate response. This limited therapeutic window is similar to that seen with neuraminidase inhibitors, which are also most effective during early stages of rapid viral replication [66] and less so once symptoms are present, particularly for children [67], [68]. Unfortunately, human exposure to an infectious agent is generally unrecognized until symptoms appear; but there are times when the risk of exposure is greatly increased. The most obvious such cases are health care professionals as well as coworkers and family members of patients. In such cases, post-exposure prophylaxis with EP67 should be within the window of window of therapeutic efficacy. Furthermore, the experiments herein used only a single dose of EP67 given at early stages of infection. It remains to be determined how multiple doses of EP67 impact the course of disease. These studies will be particularly important in the context of lethal infection or in the presence of underlying immunodeficiency.

EP67 treatment during the early stage of infection was associated with a significant reduction in viral burden and protection from morbidity/mortality. Despite the reduced viral burden, the protective humoral response to the infection was not compromised. Although there was a possible trend towards decreased IgG1, there was no reduction in the concentrations of either IgG2b or IgG2c (the homolog of IgG2a found in C57BL/6 mice) in the groups treated with EP67 compared to the non-treated control animals. Members of the IgG2 subclass are the most potent mediators of influenza virus neutralization *in vitro* and elimination *in vivo* [69]. A decrease in these critical immunoglobulin subtypes could manifest itself as impaired humoral protection. Long term protective immunity is also critically dependent upon establishment of a robust acquired immune response. Therefore, maintenance of these critical immunoglobulin subtypes following EP67 treatment indicates that induction of the early, robust innate response does not have a deleterious effect on humoral protection. Further studies to uncover the mechanism of robust humoral immunity despite the decreased viral burden, as well as the effect on CD4 and CD8 cell function following EP67 treatment, are ongoing.

The overall role of the innate immune response is to provide defense against pathogens that is both immediate and non-specific. Expanding upon the results shown herein, the response generated by EP67 in the airway should display little pathogen specificity, and thus activating the non-specific innate immune response with EP67 should prove efficacious in treating diverse viral, bacterial, and fungal infections. Beyond the promiscuity of their protection, therapeutics that operate via the innate immune response minimize mutational pressures on pathogens, since the therapeutic effect is neither directed toward nor imposed directly upon the pathogen [70].

These studies were designed to determine whether EP67, previously used primarily as an adjuvant, could provide direct protection in the face of respiratory infection. The results indicate that the response-selective stimulation of CD88-bearing effector cells in the airways provides immediate protection even from established infection, without compromising the subsequent acquired response to infection. More broadly, the results indicate that EP67 may function as a broad-spectrum emergency therapeutic for diverse respiratory infection. Ideally, an emergency therapeutic for infectious disease should exhibit several characteristics. It should provide protection when administered either prior to infection (prophylactic protection) or after infection has been established (therapeutic efficacy), and it should provide protection against multiple, diverse pathogens. In the current report, we show that EP67 fulfills the first two characteristics. Because protection was mediated via the innate immune response, EP67-induced protection should prove efficacious against diverse infectious agents, including non-viral pathogens as well as pathogens not confined to the respiratory tract. This is supported by our findings that EP67 induces effective innate immunity against both bacterial [28] and fungal (Phillips, unpublished) infection.

The peptide sequence of EP67 (YSFKDMP(MeL)aR) was originally derived from the biologically active C-terminal sequence of human C5a (C5a_65–74_: ISHKDMQLGR, 40% identity). Despite the efficacy shown in the current study, EP67 bears very little sequential similarity to the same region of murine C5a (SPHKPVQLGR). That EP67 binds to and engages C5aRs on both human and murine antigen presenting cells reflects more on the similarities of the secondary binding pocket within these C5aRs that accommodate the unique topochemical features of EP67 than it does sequential similarities of the C-terminal region of the murine and human C5a ligand.

In summary, this report shows that the C5a agonist peptide EP67 provides both prophylactic and therapeutic protection against influenza infection. Protection results from the rapid induction of a robust innate immune response that includes high local concentrations of anti-viral cytokines and the influx of several populations of innate immune effector cell types. These results have profound implications for influenza therapeutic development and, ultimately, for broad-spectrum emergency therapy against unidentified respiratory pathogens.

## Supporting Information

Figure S1 **Mice were insufflated with 30 µg of the negative control peptide scrambled EP67 ([MeL]RMYKPaFDS) in a volume of 30 µl, or with an equal volume of saline.** BAL was isolated one day later and stained for FACS analysis with CD45.2, CD11c and CD11b as described in Materials and Methods.
(TIF)
