# Supplementary figures and images for: Innate Immune Induction and Influenza Protection Elicited by a Response-Selective Agonist of Human C5a
Source: PLoS One. 2012 Jul 6;7(7):e40303. doi: 10.1371/journal.pone.0040303 (PMC3391237; doi:10.1371/journal.pone.0040303)

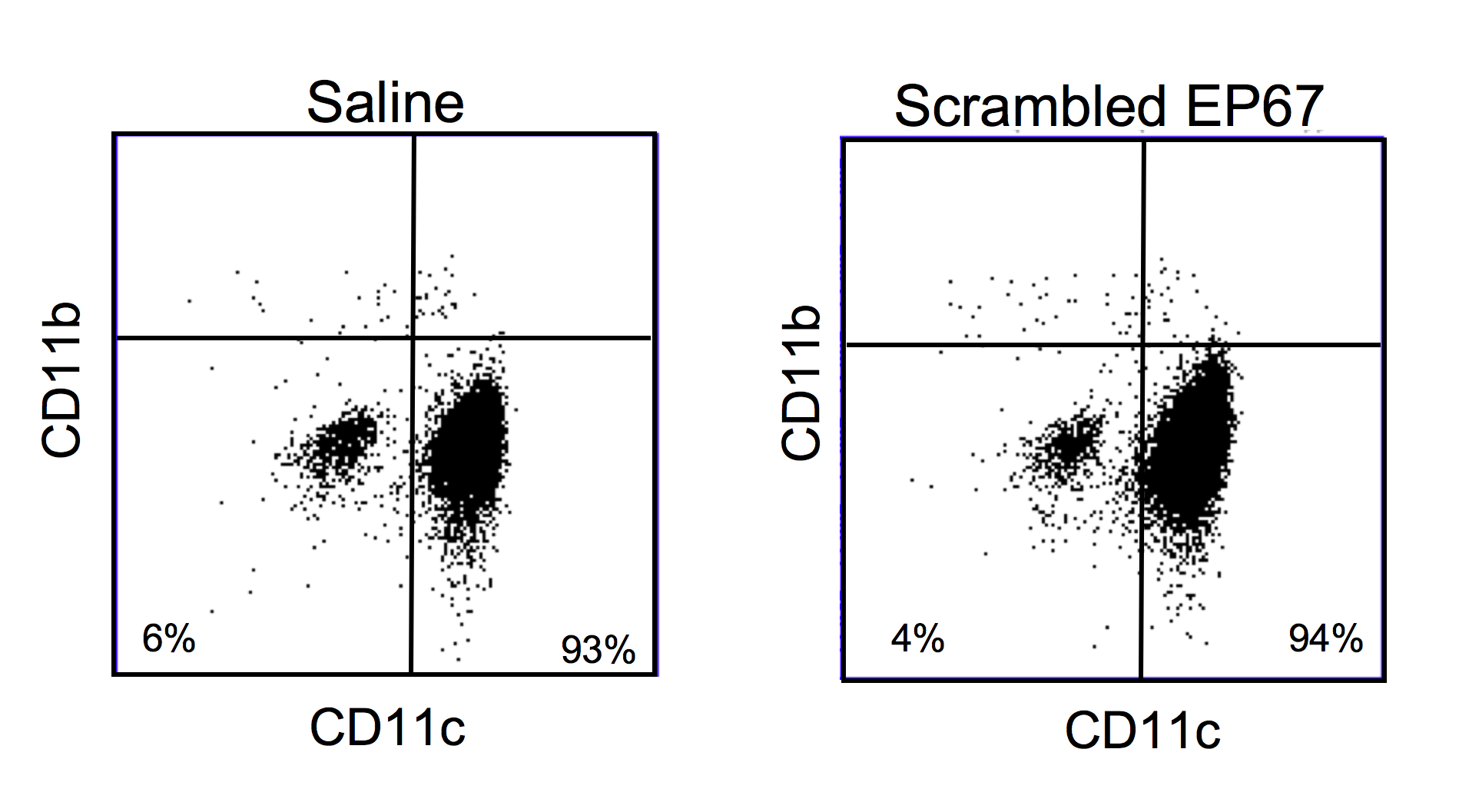

Supplement: Figure S1 — Mice were insufflated with 30 µg of the negative control peptide scrambled EP67 ([MeL]RMYKPaFDS) in a volume of 30 µl, or with an equal volume of saline. BAL was isolated one day later and stained for FACS analysis with CD45.2, CD11c and CD11b as described in Materials and Methods. (TIF) [file pone.0040303.s001.tif]
